# Dementia Is Associated With Adverse Outcomes After Nonruptured Abdominal Aortic Aneurysm Repair

**DOI:** 10.1097/AS9.0000000000000714

**Published:** 2026-09-17

**Authors:** Samir K. Shah, Lingwei Xiang, Gilbert R Upchurch, Rachel R Adler, Khanjan B Shah, Clancy J. Clark, Emily Finlayson, Dae Hyun Kim, Kueiyu Joshua Lin, Stuart R Lipsitz, Joel S. Weissman

**Affiliations:** From the * Division of Vascular Surgery, University of Florida, Gainesville, FL; † Center for Surgery and Public Health, Brigham and Women’s Hospital, Boston, MA; ‡ Division of Cardiology, University of Florida, Gainesville, FL; § Division of Surgical Oncology, Virginia Mason, Seattle, WA; ‖ Department of Surgery, Phillip R. Lee Institute for Health Policy Studies, University of California, San Francisco, CA; ¶ Marcus Institute for Aging Research, Hebrew SeniorLife, Boston, MA; # Division of Pharmacoepidemiology and Pharmacoeconomics, Department of Medicine, Brigham and Women’s Hospital, Boston, MA.

**Keywords:** abdominal aortic aneurysm, endovascular aortic aneurysm repair, dementia, surgical outcomes, Medicare claims

## Abstract

**Objective:**

To compare outcomes after nonruptured abdominal aortic aneurysm (AAA) repair among Medicare beneficiaries with and without Alzheimer disease and related dementias (ADRD).

**Background:**

AAA repair carries substantial risks; outcomes data in patients with ADRD are extremely limited.

**Methods:**

We conducted a retrospective cohort study using Medicare fee-for-service claims (2016–2020) of beneficiaries ≥66 years who underwent nonruptured AAA repair. ADRD was ascertained using a validated 12-month lookback algorithm. Outcomes were 30-, 90-day, and 1-year mortality, readmissions, major inpatient complications, and discharge destination. Associations were estimated with propensity score–weighted models adjusted for age, sex, race, Elixhauser comorbidity index, claims-based frailty, and repair type (endovascular vs open).

**Results:**

Of 42,733 repairs, 1817 (4.3%) involved patients with ADRD, who were older (median 80.8 vs 75.8 years), more often female (30.7% vs 25.0%), less often non-Hispanic White (84.2% vs 90.1%), and more likely to receive endovascular repair (89.1% vs 83.8%). Unadjusted outcomes were worse across mortality, readmissions, and major complications. After adjustment, ADRD was associated with higher mortality (30-day adjusted odds ratio [aOR] 1.50, 95% confidence interval [CI] 1.15–1.95; 1-year aOR 1.83, 95% CI 1.59–2.11), greater readmissions (30-day aOR 1.26, 95% CI 1.06–1.49; 90-day aOR 1.25, 95% CI 1.07–1.45), and increased discharge to higher-level care (aOR 2.46, 95% CI 2.12–2.85).

**Conclusions:**

In this national cohort, ADRD was associated with increased mortality, readmissions, and discharge to higher-level care after AAA repair despite more frequent use of endovascular repair.

## INTRODUCTION

Abdominal aortic aneurysm (AAA) is a common, potentially lethal, and resource-intensive vascular condition. Approximately 1.1 million Americans are living with AAA, and the disease is responsible for more than 10,000 deaths annually in the United States.^1^ At the same time, Alzheimer disease and related dementias (ADRD) are increasing in prevalence and together are a frequent comorbid condition in patients undergoing AAA treatment. Currently, nearly 6 million Americans live with dementia, a number projected to grow by more than 40% in the next decade.^2^

Dementia is associated with a wide array of adverse outcomes after surgery—including increased mortality, complications, delirium, prolonged hospitalization, and functional decline—across diverse procedures such as colorectal surgery, total knee arthroplasty, and lower extremity amputation.^3–5^ Understanding outcomes of AAA repair in patients with dementia is essential given that elective repair is intended to be life-extending rather than for immediate improvement in quality of life and because the tradeoffs between open and minimally invasive endovascular aortic aneurysm repair (EVAR) are substantial. Granular outcomes data directly support shared decision-making and help patients and families make treatment choices aligned with their healthcare goals. To this end, we sought to study AAA repair in a national population of FFS Medicare beneficiaries, focusing on the relative frequency of open and endovascular repair and associated clinical outcomes in those with and without dementia.

## METHODS

This study was approved by the Mass General Brigham institutional review board; the need for informed consent was waived.

### Data Sources and Study Population

We examined Medicare fee-for-service (FFS) claims from January 1, 2016, to December 31, 2020, and identified patients undergoing open or EVAR using *International Classification of Diseases*, 10th revision, procedure and diagnosis codes (Supplemental Table 1, https://links.lww.com/AOSO/A676) from January 1, 2017, to October 2, 2020. We excluded patients with ruptured AAA using ICD-10 diagnosis codes. Only beneficiaries 66 years and older at the time of surgery were included in order to allow for a 1-year lookback period. Beneficiaries were allowed to have up to a 1-month gap in the lookback period; all others were removed from the final cohort.

We used Medicare FFS inpatient, outpatient, carrier, home health agency, durable medical equipment, hospice, Minimum Data Set, and skilled nursing facility claims from 2016 to 2018 in addition to MedPAR from 2019 to 2020.

### Covariates and Outcomes

We used the Medicare Beneficiary Summary File to collect demographic data. We classified race/ethnicity based on the Research Triangle Institute race code.^6^ Admission source and type were used to identify urgent/emergent surgery status. Comorbid conditions were identified based on a 1-year lookback in the above-listed files and summarized using the in-hospital mortality modification of the Elixhauser comorbidity index.^7^ We defined ADRD using a validated list of ICD-10-CM codes (Supplemental Table 2, https://links.lww.com/AOSO/A676) with a sensitivity of 68.0%, specificity of 93.5%, positive predictive value of 77.1%, and negative predictive value of 90.1%.^8^

The claims-based frailty index (cFI) is derived from 93 variables based on ICD diagnosis, CPT-4, and HCPCS codes and has been validated against outcomes including mortality, mobility impairment, and disability, as well as clinical frailty measures.^9,10^ We dichotomized frailty using the cutoff of ≥0.250 to represent mild versus moderate-to-severe frailty.^9^

We measured inpatient major complications (Supplemental Table 3, https://links.lww.com/AOSO/A676), mortality and readmissions at 30 and 90 days, 1-year mortality, discharge home and to a higher level of care, 90-day intensive interventions, and 90-day time-at-home ratio. Discharge home was calculated only for community-dwelling patients, who were defined as having no Minimum Data Set assessment within 180 days before AAA. Discharge to a higher level of care was defined using the following hierarchy: home > subacute nursing facility > long-term acute care (LTAC). Acute rehabilitation centers were clustered with subacute nursing facilities. Patients coming from an LTAC were excluded for this outcome. Intensive interventions were defined as cardiopulmonary resuscitation, tracheostomy, extracorporeal membrane oxygenation, prolonged renal replacement therapy, prolonged intubation, or feeding tube insertion. Time-at-home ratio is an emerging patient-centered outcome and was calculated only for community-dwelling patients using the ratio of the number of days spent at home following the index surgery and the total follow-up days after the index surgery (maximum = 90 days). Days spent at home were calculated by subtracting the number of days spent in the hospital, hospice, skilled nursing facility, and LTAC facilities from the follow-up days.

### Statistical Analysis

Descriptive statistics for continuous variables included means, medians, interquartile ranges, and standard errors; categorical variables were described using frequencies and percentages. Group differences in unadjusted outcomes were tested using the Mann–Whitney *U* test for continuous variables and the chi-squared or Fisher exact test for categorical variables. To adjust for confounding, we first calculated inverse-propensity weighting (IPW) weights using a logistic regression model predicting the likelihood of having ADRD. Covariates in the model included age at surgery, sex, race, Elixhauser in-hospital mortality index, dual Medicare/Medicaid eligibility, frailty, metropolitan versus non-metropolitan patient residency, admission status (elective vs urgent/emergent), and repair type (EVAR vs open). Next, we used IPW-weighted generalized estimating equation linear regression models to assess continuous outcomes, including the time-at-home ratio and hospital length of stay. We then applied IPW-weighted generalized estimating equation logistic regression models for dichotomous outcomes, including mortality, readmission, discharge home, complications, intensive interventions, and discharge to a higher level of care. In addition, we evaluated the interaction between admission status (elective vs urgent/emergent) and ADRD on all outcomes and performed stratified analyses by admission status. These analyses were repeated with stratification by repair type. In stratified analyses, we conducted multivariable models adjusting for the same covariates used in the IPW weights calculation, excluding the stratification variable. Hospital clustering is accounted for in all models. All analyses were performed using SAS 9.4 (SAS, Cary, NC).

## RESULTS

### Cohort Characteristics

**FIGURE 1.**
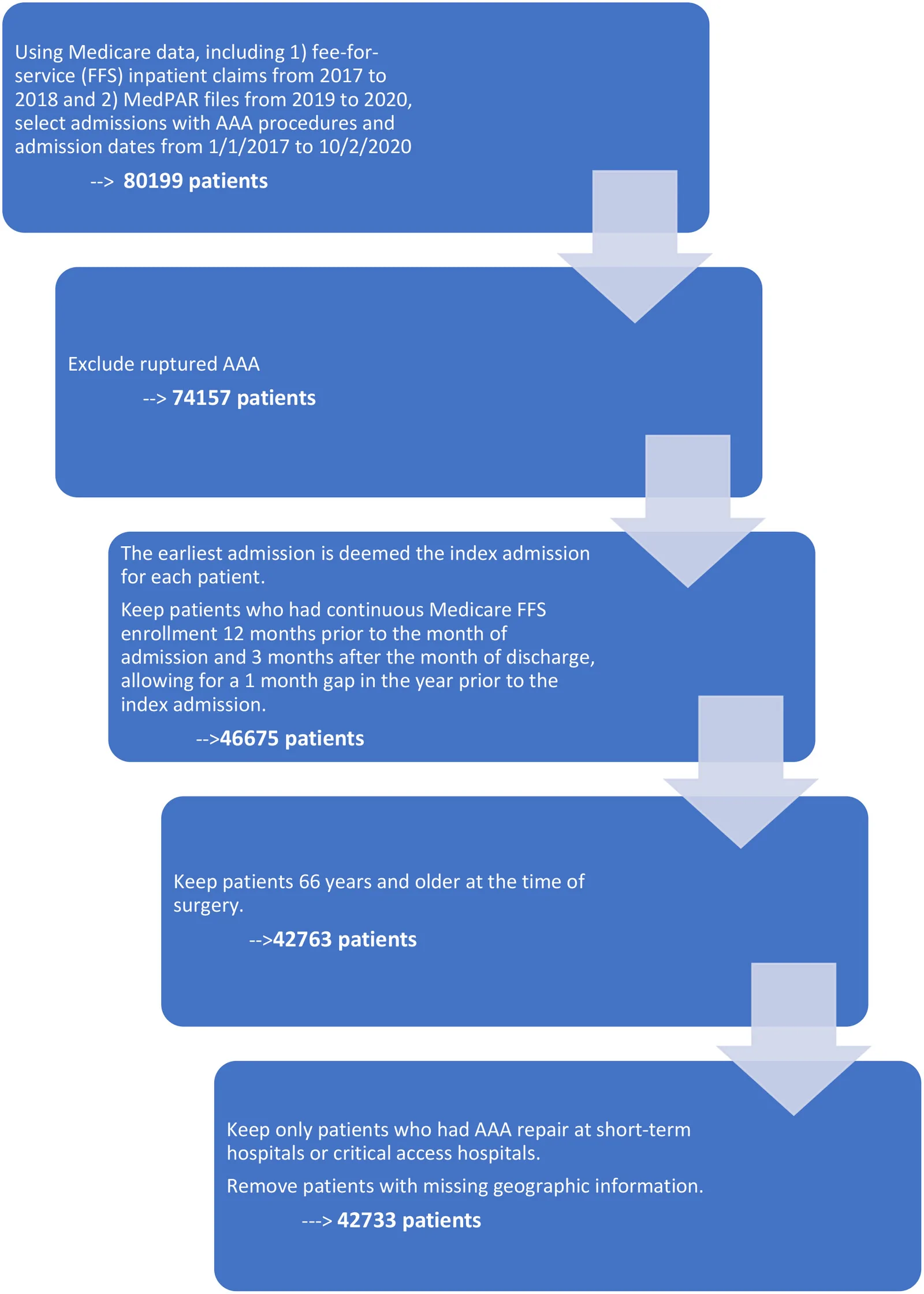
Cohort creation flow diagram.

We identified 42,733 unique AAA repairs (Fig. 1), including 1817 (4.3%) in patients with ADRD (Table 1). The median age of the overall cohort was 76.0 years. Compared with the non-ADRD cohort, the ADRD group was older (median 80.8 vs 75.8 years, *P* < 0.001), more likely to be female (30.7% vs 25.0%, *P* < 0.001), and less likely to be non-Hispanic White (84.2% vs 90.1%, *P* < 0.001). Overall, 95.9% of patients were community-dwelling, but only 78.5% of patients living with ADRD were community-dwelling. Substantially more patients with ADRD were dual Medicare/Medicaid eligible than those without ADRD: 29.1% versus 12.1% (*P* < 0.001). The 3 most common comorbid conditions in the entire cohort were uncomplicated diabetes (19.9%), heart failure (17.0%), and complicated hypertension (36.4%)—all were significantly more common (*P* < 0.001) in the ADRD group. The overall burden of comorbidities measured by the Elixhauser in-hospital mortality index was higher in patients with ADRD: median 8.0 versus 4.0 (*P* < 0.001). Similarly, moderate/severe frailty was more common in the ADRD group (38.9% vs 10.5%, *P* < 0.001).

**TABLE 1.** Patient and Procedural Characteristics by Dementia Status

		All (N = 42,733)		ADRD				
				No (N = 40,916; 95.7%)		Yes (N = 1817; 4.3%)		
		Median	IQR	Median	IQR	Median	IQR	*P*
Age		76.0	(71.3, 81.3)	75.8	(71.2, 81.0)	80.8	(75.7, 85.1)	<0.001
Elixhauser in-hospital mortality index		4.0	(0, 13)	4.0	(0, 13)	8.0	(1, 21)	<0.001
		**N**	**%**	**N**	**%**	**N**	**%**	
AAA procedure type	EVAR	35,897	84.0	34,278	83.8	1619	89.1	<0.001
	Open	6836	16.0	6,638	16.2	198	10.9	
Sex	Male	31,950	74.8	30,691	75.0	1259	69.3	<0.001
Race	Non-Hispanic White	38,379	89.8	36,849	90.1	1530	84.2	<0.001
	Black	1822	4.3	1682	4.1	140	7.7	
	Hispanic	1093	2.6	1012	2.5	81	4.5	
	Asian/Native American	690	1.6	650	1.6	40	2.2	
	Other	269	0.6	‡	‡	‡	‡	
	Unknown	480	1.1	‡	‡	‡	‡	
Patient dwelling*	Community Dwelling	25,911	95.9	24,630	97.0	1281	78.5	<0.001
Patient geographic location	Metropolitan	31,287	73.2	29,900	73.1	1387	76.3	0.002
Dual eligibility		5460	12.8	4932	12.1	528	29.1	<0.001
Frail†		4985	11.7	4278	10.5	707	38.9	<0.001
Admission urgency	Urgent/Emergent	5714	13.4	5320	13.0	394	21.7	<0.001
Uncomplicated diabetes		8504	19.9003	8031	19.6280	473	26.0319	<0.001
Heart failure		7248	16.9611	6785	16.5828	463	25.4816	<0.001
Complicated hypertension		15,575	36.4472	14,618	35.7269	957	52.6692	<0.001
Chronic lung disease		18,984	44.4247	18,073	44.1710	911	50.1376	<0.001
Severe renal disease		1798	4.2075	1653	4.0400	145	7.9802	<0.001

* Analysis restricted to patients who underwent surgery during 01/01/2017 to 12/31/2018.

† Dementia coefficients removed from calculation.

‡ Cell suppressed according to CMS data policy.

Overall, 5714 (13.4%) procedures were urgent/emergent: 394 (21.7%) among those with ADRD versus 5320 (13.0%) among those without ADRD (*P* < 0.001). EVAR was the most common treatment strategy overall (35,897, 84.0%) and more common in the ADRD cohort (1619, 89.1% vs 34,278, 83.8%, *P* < 0.001).

### Overall Outcomes

All adverse outcomes were more common in patients living with ADRD, including mortality as an inpatient (3.6% vs 2.5%, *P* = 0.003), at 30 days (6.9% vs 3.6%, *P* < 0.001), and at 90 days (12.7 vs 5.9%, *P* < 0.001) (Table 2). Major inpatient complications occurred in 23.5% of the ADRD cohort compared with 17.9% of the non-ADRD cohort (*P* < 0.001). Mean hospital length of stay was 4.4 days among patients with ADRD compared with a mean of 3.5 days among those without ADRD (*P* < 0.001). Intensive interventions at 90 days occurred in 4.9% of those with ADRD compared with 3.3% among those without ADRD (*P* < 0001). Discharge to a higher level of care was more common among those with ADRD (32.6 vs 11.0%, *P* < 0.001), and discharge to home (calculated only for community-dwelling beneficiaries) was correspondingly lower (69.5% vs 86.0%, *P* < 0.001). Overall, 20.2% of beneficiaries were readmitted at 90 days, consisting of 30.0% versus 19.7% (*P* < 0.001) among those with and without ADRD. Time-at-home ratios in the ADRD and non-ADRD cohorts were 0.76 and 0.87 (*P* < 0.001).

**TABLE 2.** Unadjusted and Adjusted Outcomes After AAA Repair by Dementia Status

	Unadjusted							Adjusted	
	All		ADRD						
			0		1				
	N	%	N	%	N	%	*P*	OR (95% CI) (Ref ADRD=0)	*P*
Inpatient death	1092	2.6	1026	2.5	66	3.6	0.003	1.13 (0.78–1.63)	0.52
30-d death	1582	3.7	1456	3.6	126	6.9	<0.001	1.50 (1.15–1.95)	0.002
90-d death	2624	6.1	2394	5.9	230	12.7	<0.001	1.59 (1.31–1.93)	<0.001
1-yr death*	4378	12.8	3878	11.9	500	28.2	<0.001	1.83 (1.59 – 2.11)	<0.001
Inpatient complication	7738	18.1	7311	17.9	427	23.5	<0.001	1.22 (1.03–1.44)	0.02
30-d complication†	9840	23.2	9267	22.8	573	32.1	<0.001	1.28 (1.10–1.49)	0.001
Discharge to higher level of care‡	4,32	11.9	4204	11.0	528	32.6	<0.001	2.46 (2.12–2.85)	<0.001
Discharge to home§	22,065	85.2	21,175	86.0	890	69.5	<0.001	0.52 (0.44–0.61)	<0.001
30-d readmission†	4785	11.6	4484	11.3	301	17.7	<0.001	1.26 (1.06–1.49)	0.007
90-d readmission†	8273	20.2	7770	19.7	503	30.0	<0.001	1.25 (1.07–1.45)	0.004
90-d any intensive intervention†	1383	3.4	1,303	3.3	80	4.9	<0.001	1.27 (0.92–1.77)	0.15
	**Mean**	**SE**	**Mean**	**SE**	**Mean**	**SE**	***P***	**Mean difference (95% CI**)	***P***
Hospital length of stay	3.57	0.03	3.54	0.03	4.35	0.15	<0.001	0.41 (0.12–0.71)	0.006
Home–time ratio‡	0.87	0.00	0.87	0.00	0.76	0.01	<0.001	−0.07 (−0.09 to −0.05)	<0.001

Covariates in the model included age, sex, race, Elixhauser in-hospital mortality index, dual Medicare/Medicaid eligibility, frailty, metropolitan versus non-metropolitan patient residency, admission status (elective vs urgent/emergent), and repair type (EVAR vs open).

* Patients who underwent index surgery before 1/1/2020 and had continuous Medicare FFS enrollment for 12 months after discharge.

† Patients who died within the follow-up time frame and without an event of interest were removed from analysis.

‡ Patients admitted from the highest level of care (LTAC) were removed from analysis.

§ Analysis was restricted to community-dwelling patients with an index admission date between 1/1/2017 and 12/31/2018.

After adjustment, these findings remained significant in nearly all cases. Patients with ADRD were more likely to have mortality at 30 (adjusted odds ratio [aOR] 1.50, 95% confidence interval [CI] 1.15–1.95) and 90 days (aOR 1.59, 95% CI 1.31–1.93), and 1-year (aOR 1.83, 95% CI 1.59–2.11). Major inpatient complications were also more likely (aOR 1.22, 95% CI 1.03–1.44) (Table 2). Discharge to a higher level of care (aOR 2.46, 95% CI 2.12–2.85) and discharge to home (aOR 0.52, 95% CI 0.44–0.61) were less favorable for those with ADRD. Patients with ADRD were more likely to be readmitted at 30 (aOR 1.26, 95% CI 1.06–1.49) and 90 days (aOR 1.25, 95% CI 1.07–1.45). The time at home ratio was worse among patients with ADRD (mean difference −0.07, 95% CI −0.09 to −0.05).

We noted 2 outcomes where unadjusted differences that were significant between the ADRD and non-ADRD cohorts became nonsignificant after adjustment. These included inpatient mortality (aOR 1.13, 95% CI 0.78–1.63) and intensive interventions at 90 days (aOR 1.27, 95% CI 0.92–1.77) (Table 2). After adjustment, hospital length of stay among those with ADRD was longer, with a mean difference of 0.41 (95% CI 0.12–0.71).

### Outcomes Stratified by Repair Type

For EVAR, patients with ADRD had higher 30- and 90-day mortality (aOR 1.55, 95% CI 1.22–1.98 and aOR 1.46, 95% CI 1.20–1.76), more 30-day complications (aOR 1.20, 95% CI 1.05–1.37), higher odds of discharge to a higher level of care (aOR 2.72, 95% CI 2.34–3.16) and lower odds of discharge home (aOR 0.45, 95% CI 0.38–0.53), longer length of stay (mean difference 0.26 95% CI 0.04–0.48), greater readmissions at 30 and 90 days (90 days: aOR 1.13, 95% CI 1.00–1.29), and a lower 90-day home–time ratio (mean difference −0.07, 95% CI −0.09 to −0.06) (Table 3). Inpatient death and inpatient complications were similar between the ADRD and non-ADRD cohorts. By contrast, among open repairs (Table 4), there were no differences that persisted after adjustment except for discharge to higher care (aOR 1.55, 95% CI 1.02–2.20) and discharge to home (aOR 0.65, 95% CI 0.45-0.94), which were worse among those with ADRD.

**TABLE 3.** Unadjusted and Adjusted Outcomes After Endovascular AAA Repair (EVAR) by Dementia Status

	Unadjusted							Adjusted	
	All (N= 35,897)		ADRD						
			No (N= 34,278)		Yes (N = 1619)				
	N	%	N	%	N	%	*P*	OR (95% CI) (Ref ADRD = 0)	*P*
Inpatient death	484	1.3	436	1.3	48	3.0	<0.001	1.35 (0.97–1.86)	0.07
30-d death	878	2.4	776	2.3	102	6.3	<0.001	1.55 (1.22–1.98)	<0.001
90-d death	1703	4.7	1513	4.4	190	11.7	<0.001	1.46 (1.20–1.76)	<0.001
1-yr death*	3328	11.6	2885	10.6	443	28.0	<0.001	1.79 (1.57 – 2.05)	<0.001
Inpatient complication	4117	11.5	3808	11.1	309	19.1	<0.001	1.12 (0.96–1.29)	0.15
30-d complication†	5962	16.7	5513	16.2	449	28.3	<0.001	1.20 (1.05–1.37)	0.007
Discharge to higher level of care‡	2808	8.3	2373	7.3	435	29.9	<0.001	2.72 (2.34–3.16)	<0.001
Discharge to home§	19,454	90.6	18,619	91.6	835	73.5	<0.001	0.45 (0.38–0.53)	<0.001
30-d readmission†	3747	10.7	3480	10.4	267	17.5	<0.001	1.18 (1.01–1.37)	0.04
90-d readmission†	6713	19.2	6266	18.7	447	29.6	<0.001	1.13 (1.00–1.29)	0.05
90-d any intensive intervention†	616	1.8	560	1.7	56	3.8	<0.001	1.27 (0.93–1.74)	0.14
	**Mean**	**SE**	**Mean**	**SE**	**Mean**	**SE**	***P***	**Mean Difference (95% CI**)	***P***
Hospital length of stay	2.45	0.02	2.40	0.02	3.50	0.11	<0.001	0.26 (0.04–0.48)	0.02
Home–time ratio§	0.91	0.00	0.91	0.00	0.79	0.01	<0.001	−0.07 (−0.09 to −0.06)	<0.001

* Patients who underwent index surgery before 1/1/2020 and had continuous Medicare FFS enrollment for 12 months after discharge.

† Patients who died within the follow-up time frame and without an event of interest were removed from analysis.

‡ Patients admitted from the highest level of care (LTAC) were removed from analysis.

§ Analysis was restricted to community-dwelling patients with an index admission date between 1/1/2017 and 12/31/2018.

**TABLE 4.** Unadjusted and Adjusted Outcomes After Open AAA Repair by Dementia Status

	Unadjusted							Adjusted	
	All (N = 6836)		ADRD						
			No (N = 6638)		Yes (N = 198)				
	N	%	N	%	N	%	*P*	OR (95% CI) (Ref ADRD = 0)	*P*
Inpatient death	608	8.9	590	8.9	18	9.1	0.92	0.66 (0.39–1.13)	0.13
30-d death	704	10.3	680	10.2	24	12.1	0.39	0.76 (0.47–1.22)	0.26
90-d death	921	13.5	881	13.3	40	20.2	0.005	1.04 (0.68–1.58)	0.85
1-yr death*	1050	19.1	993	18.7	57	30.2	<0.001	1.13 (0.77–1.65)	0.54
Inpatient complication	3621	53.0	3503	52.8	118	59.6	0.06	0.94 (0.68–1.30)	0.72
30-d complication†	3878	57.3	3754	57.2	124	63.6	0.07	0.93 (0.66–1.30)	0.66
Discharge to higher level of care‡	1924	33.6	1831	32.9	93	57.1	<0.001	1.55 (1.09–2.20)	0.01
Discharge to home§	2611	58.8	2556	59.5	55	37.9	<0.001	0.65 (0.45–0.94)	0.02
30-d readmission†	1038	17.0	1004	17.0	34	19.8	0.33	0.92 (0.64–1.32)	0.64
90-d readmission*	1560	25.8	1504	25.5	56	33.3	0.03	1.02 (0.73–1.43)	0.90
90-d any intensive intervention*	767	12.2	743	12.1	24	13.7	0.53	0.71 (0.44–1.15)	0.17
	**Mean**	**SE**	**Mean**	**SE**	**Mean**	**SE**	***P***	**Mean difference (95% CI**)	***P***
Hospital length of stay	9.46	0.11	9.40	0.11	11.28	0.97	0.04	0.33 (−1.48–2.13)	0.72
Home–time ratio§	0.68	0.01	0.69	0.01	0.54	0.03	<0.001	−0.06 (−0.12–0.01)	0.07

* Patients who underwent index surgery before 1/1/2020 and had continuous Medicare FFS enrollment for 12 months after discharge.

† Patients who died within the follow-up time frame and without an event of interest were removed from analysis.

‡ Patients admitted from the highest level of care (LTAC) were removed from analysis.

§ Analysis was restricted to community-dwelling patients with an index admission date between 1/1/2017 and 12/31/2018.

¶ Analysis was restricted to community-dwelling patients and those discharged before 10/1/2018. And patients who died within the follow-up time frame and without event of interest were removed from analysis.

### Outcomes Stratified by Admission Status

The interaction between admission status and ADRD was significant for 90-day mortality (*P* = 0.04) but not for 30-day or inpatient mortality. Among elective cases (N = 37,019), ADRD was associated with higher 30-day mortality (aOR 1.35, 95% CI 1.01–1.79), 90-day mortality (aOR 1.52, 95% CI 1.23–1.88), 1-year mortality (aOR 1.86, 95% CI 1.61 – 2.14), 30-day complications (aOR 1.24, 95% CI 1.08–1.43), discharge to higher level of care (aOR 2.82, 95% CI 2.42–3.30), greater readmissions at 30 and 90 days (90 days: aOR 1.21, 95% CI 1.06-1.39), lower discharge home (aOR 0.48, 95% CI 0.40–0.58), and a lower 90-day home-time ratio (mean difference −0.06, 95% CI −0.08 to −0.05). Among urgent/emergent cases (N = 5714), ADRD was associated with discharge to higher level of care (aOR 1.98, 95% CI 1.53–2.56), lower discharge home (aOR 0.45, 95% CI 0.33–0.60), and a lower 90-day home–time ratio (mean difference −0.08, 95% CI −0.13 to −0.04), but mortality differences were not significant. Full results are presented in Supplemental Table 4, https://links.lww.com/AOSO/A676.

## DISCUSSION

Our data show that AAA repair in patients living with ADRD is associated with a spectrum of poor outcomes despite preferential use of EVAR. On adjusted analysis, ADRD was associated with approximately 1.5 higher odds of 30- and 90-day mortality, major inpatient complications, and nearly three-fold greater need for higher-level post-acute institutional care. Most strikingly, more than 1 in 4 patients with ADRD died within 1 year of repair.

The association between ADRD and adverse outcomes was largely driven by EVAR, which comprised most procedures in our cohort. In our stratified analyses, among EVAR cases, ADRD remained independently associated with more 30-day complications, higher 30- and 90-day mortality, greater 30- and 90-day readmission risk, markedly higher odds of discharge to a higher level of care, and lower odds of discharge home.

By contrast, among open repairs, there were no significant adjusted differences among beneficiaries with and without ADRD other than higher odds of discharge to a higher level of care and lower likelihood of discharge home for the former. Given the predominance of EVAR use among patients with ADRD (89.1%), the EVAR-specific risks likely account for most of the overall associations we observe between ADRD and worse outcomes after AAA repair. This makes our results even more striking given the common assumption that EVAR is a less invasive, shorter, and less physiologically stressful operation than open repair.

When stratified by admission status, the adverse associations of ADRD with mortality and complications were concentrated among elective cases, where 30- and 90-day mortality remained significantly elevated. In the urgent/emergent subgroup, only discharge disposition outcomes remained significant. This pattern may reflect selection effects—patients with ADRD undergoing urgent/emergent repair may represent a narrower clinical spectrum—but it underscores the importance of preoperative shared decision-making in the elective setting, where the decision to proceed with repair is most modifiable.

Our data are novel—we identified no studies focused exclusively on AAA repair outcomes in patients living with dementia—and confirm and extend prior analyses by isolating AAA repair and quantifying dementia-specific risk. Several studies that examined AAA repair outcomes in patients with dementia as part of a larger group of surgeries (eg, all vascular surgery or all high-risk surgery) supported our findings.^11,12^ The largest of such studies included 1860 aortic aneurysm repairs of all types in patients living with dementia in a larger group of 27,920 patients undergoing vascular surgeries.^12^ This analysis similarly showed increased 30- and 90-day mortality, longer hospital length of stay, worse likelihood of discharge to home, and time-at-home ratio. Unlike our current study, it showed no increase in inpatient complications, but these results were for the entire group of surgeries, not AAA alone, and included a variety of other vascular surgeries, such as limb amputation and peripheral arterial surgery.

AAA repair is almost always pursued for life extension rather than symptomatic relief. Our findings that postoperative survival is attenuated and time spent at home is reduced should prompt explicit goals-of-care conversations that weigh rupture risk against the lived experience of recovery. Frameworks such as the American College of Surgeons Geriatric Surgery Verification Program recommend preoperative cognitive and frailty screening; our data lend support to these standards for all older adults being evaluated for AAA repair given the profound impact of ADRD on outcomes.^13^

Further work should (1) develop and validate prediction models that combine aneurysm morphology, frailty, and cognitive impairment to identify patients unlikely to benefit from repair; (2) capture patient-centered outcomes such as mobility, pain, and caregiver-reported quality-of-life trajectories following repair; (3) evaluate strategies to mitigate poor outcomes such as geriatric co-management; and (4) construct and evaluate shared-decision aids tailored for patients with cognitive impairment and their surrogates.

Our study has several important limitations. We lack granular data on dementia severity, operative and anatomic details, and important patient-centered outcomes like mobility. Only 39% of patients with ADRD in our cohort met the cFI threshold for frailty (≥0.25). This apparent discordance likely reflects both the multidimensional nature of the cFI and the limitations of claims-based frailty ascertainment, in which under-coding of functional impairments may lead to underestimation of frailty burden. Furthermore, because the cFI ≥0.25 cutpoint has been shown to approximate moderate-to-severe dementia, many ADRD patients in our cohort with mild or early-stage disease would not be expected to cross this threshold.^14^ Prospective frailty assessment tools such as the Clinical Frailty Scale or Comprehensive Geriatric Assessment, which directly incorporate cognitive status, may capture this relationship more completely than claims-based measures. Our analysis is entirely based on FFS Medicare beneficiaries, and extrapolation to other populations should be done with caution. Our stratified analyses may be underpowered, especially for open repair, and selection for open surgery likely differs by cognitive status, which could bias effect estimates towards the null despite clinically important differences. Last, we lack outcomes on patients who qualified for repair anatomically but declined or were not offered repair, preventing direct comparison with nonoperative treatment.

Key strengths include the large, contemporary, nationally representative sample; focus on AAA alone rather than larger, heterogeneous groups of procedures; and use of validated frailty and ADRD definitions.

In conclusion, in a contemporary national Medicare cohort, dementia was present in 4.3% of nonruptured AAA repairs and was associated with higher 30- and 90-day and 1-year mortality, more major complications, increased readmissions, and a 2- to 3-fold greater need for higher-level post-acute institutional care despite predominant use of EVAR. These data should be used to inform decision-making for older adults with ADRD considering AAA repair. Future studies should focus on measuring patient-centered outcomes and identifying potential interventions to mitigate poor outcomes.

S.K.S. and J.S.W. contributed to the conception and design, acquisition of data, analysis, and interpretation of data, drafting of the article, critical revision, and final approval. L.X. and R.R.A. contributed to the conception and design, acquisition of data, analysis, and interpretation of data, critical revision, and final approval. G.R.U., K.B.S., C.J.C., E.F., D.H.K., K.J.L., and S.R.L. contributed to the acquisition of data, analysis, and interpretation of data, critical revision, and final approval.

## ACKNOWLEDGMENTS

The corresponding author confirms that all individuals who contributed significantly to this work have been listed as authors.

## Supplementary Material


