# Supplementary material for: Dementia Is Associated With Adverse Outcomes After Nonruptured Abdominal Aortic Aneurysm Repair
Source: Ann Surg Open. 2026 Sep 17;7(3):e714. doi: 10.1097/AS9.0000000000000714 (PMC13596955; doi:10.1097/AS9.0000000000000714)
Supplement: Supplementary file 1 [file as9-7-e714-s001.pdf]

Supplementary Material

Supplementary Table 1. ICD-10 Diagnosis and Procedure Codes for Open and Endovascular Abdominal Aortic Aneurysm Diagnosis and Repair

Repair Type	ICD-10 Code	Code Description
Open Repair		
	0410096	Bypass Abdominal Aorta to Right Common Iliac Artery with Autologous Venous Tissue, Open Approach
	0410097	Bypass Abdominal Aorta to Left Common Iliac Artery with Autologous Venous Tissue, Open Approach
	0410098	Bypass Abdominal Aorta to Bilateral Common Iliac Arteries with Autologous Venous Tissue, Open Approach
	0410496	Bypass Abdominal Aorta to Right Common Iliac Artery with Autologous Venous Tissue, Percutaneous Endoscopic Approach
	0410497	Bypass Abdominal Aorta to Left Common Iliac Artery with Autologous Venous Tissue, Percutaneous Endoscopic Approach
	0410498	Bypass Abdominal Aorta to Bilateral Common Iliac Arteries with Autologous Venous Tissue, Percutaneous Endoscopic Approach
	041009D	Bypass Abdominal Aorta to Right External Iliac Artery with Autologous Venous Tissue, Open Approach
	041009F	Bypass Abdominal Aorta to Left External Iliac Artery with Autologous Venous Tissue, Open Approach
	041009G	Bypass Abdominal Aorta to Bilateral External Iliac Arteries with Autologous Venous Tissue, Open Approach
	041009H	Bypass Abdominal Aorta to Right Femoral Artery with Autologous Venous Tissue, Open Approach
	041009J	Bypass Abdominal Aorta to Left Femoral Artery with Autologous Venous Tissue, Open Approach
	041009K	Bypass Abdominal Aorta to Bilateral Femoral Arteries with Autologous Venous Tissue, Open Approach
	041009Q	Bypass Abdominal Aorta to Lower Extremity Artery with Autologous Venous Tissue, Open Approach
	041009R	Bypass Abdominal Aorta to Lower Artery with Autologous Venous Tissue, Open Approach
	04100A6	Bypass Abdominal Aorta to Right Common Iliac Artery with Autologous Arterial Tissue, Open Approach
	04100A7	Bypass Abdominal Aorta to Left Common Iliac Artery with Autologous Arterial Tissue, Open Approach
	04100A8	Bypass Abdominal Aorta to Bilateral Common Iliac Arteries with Autologous Arterial Tissue, Open Approach
	04100AD	Bypass Abdominal Aorta to Right External Iliac Artery with Autologous Arterial Tissue, Open Approach
	04100AF	Bypass Abdominal Aorta to Left External Iliac Artery with Autologous Arterial Tissue, Open Approach
	04100AG	Bypass Abdominal Aorta to Bilateral External Iliac Arteries with Autologous Arterial Tissue, Open Approach
	04100AH	Bypass Abdominal Aorta to Right Femoral Artery with Autologous Arterial Tissue, Open Approach
	04100AJ	Bypass Abdominal Aorta to Left Femoral Artery with Autologous Arterial Tissue, Open Approach
	04100AK	Bypass Abdominal Aorta to Bilateral Femoral Arteries with Autologous Arterial Tissue, Open Approach
	04100AQ	Bypass Abdominal Aorta to Lower Extremity Artery with Autologous Arterial Tissue, Open Approach
	04100AR	Bypass Abdominal Aorta to Lower Artery with Autologous Arterial Tissue, Open Approach
	04100J6	Bypass Abdominal Aorta to Right Common Iliac Artery with Synthetic Substitute, Open Approach
	04100J7	Bypass Abdominal Aorta to Left Common Iliac Artery with Synthetic Substitute, Open Approach
	04100J8	Bypass Abdominal Aorta to Bilateral Common Iliac Arteries with Synthetic Substitute, Open Approach
	04100JD	Bypass Abdominal Aorta to Right External Iliac Artery with Synthetic Substitute, Open Approach
	04100JF	Bypass Abdominal Aorta to Left External Iliac Artery with Synthetic Substitute, Open Approach

04100JG	Bypass Abdominal Aorta to Bilateral External Iliac Arteries with Synthetic Substitute, Open Approach
04100JH	Bypass Abdominal Aorta to Right Femoral Artery with Synthetic Substitute, Open Approach
04100JJ	Bypass Abdominal Aorta to Left Femoral Artery with Synthetic Substitute, Open Approach
04100JK	Bypass Abdominal Aorta to Bilateral Femoral Arteries with Synthetic Substitute, Open Approach
04100JQ	Bypass Abdominal Aorta to Lower Extremity Artery with Synthetic Substitute, Open Approach
04100JR	Bypass Abdominal Aorta to Lower Artery with Synthetic Substitute, Open Approach
04100K6	Bypass Abdominal Aorta to Right Common Iliac Artery with Nonautologous Tissue Substitute, Open Approach
04100K7	Bypass Abdominal Aorta to Left Common Iliac Artery with Nonautologous Tissue Substitute, Open Approach
04100K8	Bypass Abdominal Aorta to Bilateral Common Iliac Arteries with Nonautologous Tissue Substitute, Open Approach
04100KD	Bypass Abdominal Aorta to Right External Iliac Artery with Nonautologous Tissue Substitute, Open Approach
04100KF	Bypass Abdominal Aorta to Left External Iliac Artery with Nonautologous Tissue Substitute, Open Approach
04100KG	Bypass Abdominal Aorta to Bilateral External Iliac Arteries with Nonautologous Tissue Substitute, Open Approach
04100KH	Bypass Abdominal Aorta to Right Femoral Artery with Nonautologous Tissue Substitute, Open Approach
04100KJ	Bypass Abdominal Aorta to Left Femoral Artery with Nonautologous Tissue Substitute, Open Approach
04100KK	Bypass Abdominal Aorta to Bilateral Femoral Arteries with Nonautologous Tissue Substitute, Open Approach
04100KQ	Bypass Abdominal Aorta to Lower Extremity Artery with Nonautologous Tissue Substitute, Open Approach
04100KR	Bypass Abdominal Aorta to Lower Artery with Nonautologous Tissue Substitute, Open Approach
04100Z6	Bypass Abdominal Aorta to Right Common Iliac Artery, Open Approach
04100Z7	Bypass Abdominal Aorta to Left Common Iliac Artery, Open Approach
04100Z8	Bypass Abdominal Aorta to Bilateral Common Iliac Arteries, Open Approach
04100ZD	Bypass Abdominal Aorta to Right External Iliac Artery, Open Approach
04100ZF	Bypass Abdominal Aorta to Left External Iliac Artery, Open Approach
04100ZG	Bypass Abdominal Aorta to Bilateral External Iliac Arteries, Open Approach
04100ZH	Bypass Abdominal Aorta to Right Femoral Artery, Open Approach
04100ZJ	Bypass Abdominal Aorta to Left Femoral Artery, Open Approach
04100ZK	Bypass Abdominal Aorta to Bilateral Femoral Arteries, Open Approach
04100ZQ	Bypass Abdominal Aorta to Lower Extremity Artery, Open Approach
04100ZR	Bypass Abdominal Aorta to Lower Artery, Open Approach
041049D	Bypass Abdominal Aorta to Right External Iliac Artery with Autologous Venous Tissue, Percutaneous Endoscopic Approach
041049F	Bypass Abdominal Aorta to Left External Iliac Artery with Autologous Venous Tissue, Percutaneous Endoscopic Approach
041049G	Bypass Abdominal Aorta to Bilateral External Iliac Arteries with Autologous Venous Tissue, Percutaneous Endoscopic Approach
041049H	Bypass Abdominal Aorta to Right Femoral Artery with Autologous Venous Tissue, Percutaneous Endoscopic Approach
041049J	Bypass Abdominal Aorta to Left Femoral Artery with Autologous Venous Tissue, Percutaneous Endoscopic Approach
041049K	Bypass Abdominal Aorta to Bilateral Femoral Arteries with Autologous Venous Tissue, Percutaneous Endoscopic Approach
041049Q	Bypass Abdominal Aorta to Lower Extremity Artery with Autologous Venous Tissue, Percutaneous Endoscopic Approach
041049R	Bypass Abdominal Aorta to Lower Artery with Autologous Venous Tissue, Percutaneous Endoscopic Approach

04104A6	Bypass Abdominal Aorta to Right Common Iliac Artery with Autologous Arterial Tissue, Percutaneous Endoscopic Approach
04104A7	Bypass Abdominal Aorta to Left Common Iliac Artery with Autologous Arterial Tissue, Percutaneous Endoscopic Approach
04104A8	Bypass Abdominal Aorta to Bilateral Common Iliac Arteries with Autologous Arterial Tissue, Percutaneous Endoscopic Approach
04104AD	Bypass Abdominal Aorta to Right External Iliac Artery with Autologous Arterial Tissue, Percutaneous Endoscopic Approach
04104AF	Bypass Abdominal Aorta to Left External Iliac Artery with Autologous Arterial Tissue, Percutaneous Endoscopic Approach
04104AG	Bypass Abdominal Aorta to Bilateral External Iliac Arteries with Autologous Arterial Tissue, Percutaneous Endoscopic Approach
04104AH	Bypass Abdominal Aorta to Right Femoral Artery with Autologous Arterial Tissue, Percutaneous Endoscopic Approach
04104AJ	Bypass Abdominal Aorta to Left Femoral Artery with Autologous Arterial Tissue, Percutaneous Endoscopic Approach
04104AK	Bypass Abdominal Aorta to Bilateral Femoral Arteries with Autologous Arterial Tissue, Percutaneous Endoscopic Approach
04104AQ	Bypass Abdominal Aorta to Lower Extremity Artery with Autologous Arterial Tissue, Percutaneous Endoscopic Approach
04104AR	Bypass Abdominal Aorta to Lower Artery with Autologous Arterial Tissue, Percutaneous Endoscopic Approach
04104J6	Bypass Abdominal Aorta to Right Common Iliac Artery with Synthetic Substitute, Percutaneous Endoscopic Approach
04104J7	Bypass Abdominal Aorta to Left Common Iliac Artery with Synthetic Substitute, Percutaneous Endoscopic Approach
04104J8	Bypass Abdominal Aorta to Bilateral Common Iliac Arteries with Synthetic Substitute, Percutaneous Endoscopic Approach
04104JD	Bypass Abdominal Aorta to Right External Iliac Artery with Synthetic Substitute, Percutaneous Endoscopic Approach
04104JF	Bypass Abdominal Aorta to Left External Iliac Artery with Synthetic Substitute, Percutaneous Endoscopic Approach
04104JG	Bypass Abdominal Aorta to Bilateral External Iliac Arteries with Synthetic Substitute, Percutaneous Endoscopic Approach
04104JH	Bypass Abdominal Aorta to Right Femoral Artery with Synthetic Substitute, Percutaneous Endoscopic Approach
04104JJ	Bypass Abdominal Aorta to Left Femoral Artery with Synthetic Substitute, Percutaneous Endoscopic Approach
04104JK	Bypass Abdominal Aorta to Bilateral Femoral Arteries with Synthetic Substitute, Percutaneous Endoscopic Approach
04104JQ	Bypass Abdominal Aorta to Lower Extremity Artery with Synthetic Substitute, Percutaneous Endoscopic Approach
04104JR	Bypass Abdominal Aorta to Lower Artery with Synthetic Substitute, Percutaneous Endoscopic Approach
04104K6	Bypass Abdominal Aorta to Right Common Iliac Artery with Nonautologous Tissue Substitute, Percutaneous Endoscopic Approach
04104K7	Bypass Abdominal Aorta to Left Common Iliac Artery with Nonautologous Tissue Substitute, Percutaneous Endoscopic Approach
04104K8	Bypass Abdominal Aorta to Bilateral Common Iliac Arteries with Nonautologous Tissue Substitute, Percutaneous Endoscopic Approach
04104KD	Bypass Abdominal Aorta to Right External Iliac Artery with Nonautologous Tissue Substitute, Percutaneous Endoscopic Approach
04104KF	Bypass Abdominal Aorta to Left External Iliac Artery with Nonautologous Tissue Substitute, Percutaneous Endoscopic Approach
04104KG	Bypass Abdominal Aorta to Bilateral External Iliac Arteries with Nonautologous Tissue Substitute, Percutaneous Endoscopic Approach
04104KH	Bypass Abdominal Aorta to Right Femoral Artery with Nonautologous Tissue Substitute, Percutaneous Endoscopic Approach
04104KJ	Bypass Abdominal Aorta to Left Femoral Artery with Nonautologous Tissue Substitute, Percutaneous Endoscopic Approach
04104KK	Bypass Abdominal Aorta to Bilateral Femoral Arteries with Nonautologous Tissue Substitute, Percutaneous Endoscopic Approach
04104KQ	Bypass Abdominal Aorta to Lower Extremity Artery with Nonautologous Tissue Substitute, Percutaneous Endoscopic Approach
04104KR	Bypass Abdominal Aorta to Lower Artery with Nonautologous Tissue Substitute, Percutaneous Endoscopic Approach
04104Z6	Bypass Abdominal Aorta to Right Common Iliac Artery, Percutaneous Endoscopic Approach

	04104Z7	Bypass Abdominal Aorta to Left Common Iliac Artery, Percutaneous Endoscopic Approach
	04104Z8	Bypass Abdominal Aorta to Bilateral Common Iliac Arteries, Percutaneous Endoscopic Approach
	04104ZD	Bypass Abdominal Aorta to Right External Iliac Artery, Percutaneous Endoscopic Approach
	04104ZF	Bypass Abdominal Aorta to Left External Iliac Artery, Percutaneous Endoscopic Approach
	04104ZG	Bypass Abdominal Aorta to Bilateral External Iliac Arteries, Percutaneous Endoscopic Approach
	04104ZH	Bypass Abdominal Aorta to Right Femoral Artery, Percutaneous Endoscopic Approach
	04104ZJ	Bypass Abdominal Aorta to Left Femoral Artery, Percutaneous Endoscopic Approach
	04104ZK	Bypass Abdominal Aorta to Bilateral Femoral Arteries, Percutaneous Endoscopic Approach
	04104ZQ	Bypass Abdominal Aorta to Lower Extremity Artery, Percutaneous Endoscopic Approach
	04104ZR	Bypass Abdominal Aorta to Lower Artery, Percutaneous Endoscopic Approach
	04500ZZ	Destruction of Abdominal Aorta, Open Approach
	04503ZZ	Destruction of Abdominal Aorta, Percutaneous Approach
	04504ZZ	Destruction of Abdominal Aorta, Percutaneous Endoscopic Approach
	04B00ZZ	Excision of Abdominal Aorta, Open Approach
	04B03ZZ	Excision of Abdominal Aorta, Percutaneous Approach
	04B04ZZ	Excision of Abdominal Aorta, Percutaneous Endoscopic Approach
	04R007Z	Replacement of Abdominal Aorta with Autologous Tissue Substitute, Open Approach
	04R00JZ	Replacement of Abdominal Aorta with Synthetic Substitute, Open Approach
	04R00KZ	Replacement of Abdominal Aorta with Nonautologous Tissue Substitute, Open Approach
	04R047Z	Replacement of Abdominal Aorta with Autologous Tissue Substitute, Percutaneous Endoscopic Approach
	04R04JZ	Replacement of Abdominal Aorta with Synthetic Substitute, Percutaneous Endoscopic Approach
	04R04KZ	Replacement of Abdominal Aorta with Nonautologous Tissue Substitute, Percutaneous Endoscopic Approach
	04U00JZ	Supplement Abdominal Aorta with Synthetic Substitute, Open Approach
	04V00Z6	Restriction of Abdominal Aorta, Bifurcation, Open Approach
	04V00ZZ	Restriction of Abdominal Aorta, Open Approach
	04V04Z6	Restriction of Abdominal Aorta, Bifurcation, Percutaneous Endoscopic Approach
	04V04ZZ	Restriction of Abdominal Aorta, Percutaneous Endoscopic Approach
Endovascular	04U03JZ	Supplement Abdominal Aorta with Synthetic Substitute, Percutaneous Approach
	04U04JZ	Supplement Abdominal Aorta with Synthetic Substitute, Percutaneous Endoscopic Approach
	04V00D6	Restriction of Abdominal Aorta, Bifurcation, with Intraluminal Device, Open Approach
	04V00DZ	Restriction of Abdominal Aorta with Intraluminal Device, Open Approach
	04V03D6	Restriction of Abdominal Aorta, Bifurcation, with Intraluminal Device, Percutaneous Approach
	04V03DZ	Restriction of Abdominal Aorta with Intraluminal Device, Percutaneous Approach
	04V04D6	Restriction of Abdominal Aorta, Bifurcation, with Intraluminal Device, Percutaneous Endoscopic Approach
	04V04DZ	Restriction of Abdominal Aorta with Intraluminal Device, Percutaneous Endoscopic Approach
Ruptured AAA	I713	Abdominal aortic aneurysm, ruptured
	I718	Aortic aneurysm of unspecified site, ruptured
Non-ruptured AAA	I714	Abdominal aortic aneurysm, without rupture

I719 Aortic aneurysm of unspecified site, without rupture

Supplementary Table 2. ICD-10 Diagnosis Codes for Alzheimer's Disease and Related Dementias

ICD-10 Code	Description
F0150	Vascular dementia without behavioral disturbance
F0151	Vascular dementia with behavioral disturbance
F0280	Dementia in other diseases classified elsewhere without behavioral disturbance
F0281	Dementia in other diseases classified elsewhere with behavioral disturbance
F0390	Unspecified dementia without behavioral disturbance
F05	Delirium due to known physiological condition
F04	Amnestic disorder due to known physiological condition
F060	Psychotic disorder with hallucinations due to known physiological condition
F061	Catatonic disorder due to known physiological condition
F068	Other specified mental disorders due to known physiological condition
G309	Alzheimer's disease, unspecified
G300	Alzheimer's disease with early onset
G301	Alzheimer's disease with late onset
G308	Other Alzheimer's disease
G3101	Pick's disease
G3109	Other frontotemporal dementia
G311	Senile degeneration of brain, not elsewhere classified
G94	Other disorders of brain in diseases classified elsewhere
G132	Systemic atrophy primarily affecting the central nervous system in myxedema
G138	Systemic atrophy primarily affecting central nervous system in other diseases classified elsewhere
G312	Degeneration of nervous system due to alcohol
G914	Hydrocephalus in diseases classified elsewhere
R4181	Age-related cognitive decline
R54	Age-related physical debility

Supplementary Table 3. ICD-10 Diagnosis Codes to Define Major Postoperative Complications

ICD-10	Long Descriptions
K22.8	Esophageal hemorrhage
T79.8XXA	Posttraumatic wound infection not elsewhere classified
I21.01	ST elevation (STEMI) myocardial infarction involving left main coronary artery
I21.02	ST elevation (STEMI) myocardial infarction involving left anterior descending coronary artery
I21.09	ST elevation (STEMI) myocardial infarction involving other coronary artery of anterior wall
I21.11	ST elevation (STEMI) myocardial infarction involving right coronary artery
I21.19	ST elevation (STEMI) myocardial infarction involving other coronary artery of inferior wall
I21.21	ST elevation (STEMI) myocardial infarction involving left circumflex coronary artery
I21.29	ST elevation (STEMI) myocardial infarction involving other sites
I21.3	ST elevation (STEMI) myocardial infarction of unspecified site
I21.4	Non-ST elevation (NSTEMI) myocardial infarction
I21.9	Acute myocardial infarction, unspecified
I21.A1	Myocardial infarction type 2
I21.A9	Other myocardial infarction type
I220	Subsequent ST elevation (STEMI) myocardial infarction of anterior wall
I221	Subsequent ST elevation (STEMI) myocardial infarction of inferior wall
I222	Subsequent non-ST elevation (NSTEMI) myocardial infarction
I228	Subsequent ST elevation (STEMI) myocardial infarction of other sites
I229	Subsequent ST elevation (STEMI) myocardial infarction of unspecified site
I26.90	Septic pulmonary embolism without acute cor pulmonale
I26.99	Other pulmonary embolism without acute cor pulmonale
T80.0XXA	Air embolism following infusion, transfusion and therapeutic injection, initial encounter
T81.718A	Complication of other artery following a procedure, not elsewhere classified, initial encounter
T81.72XA	Complication of vein following a procedure, not elsewhere classified, initial encounter
T8172XD	Complication of vein following a procedure, not elsewhere classified, subsequent encounter
T8172XS	Complication of vein following a procedure, not elsewhere classified, sequela
T81719A	Complication of unspecified artery following a procedure, not elsewhere classified, initial encounter
T81719D	Complication of unspecified artery following a procedure, not elsewhere classified, subsequent encounter
T81719S	Complication of unspecified artery following a procedure, not elsewhere classified, sequela
T82.817A	Embolism due to cardiac prosthetic devices, implants and grafts, initial encounter
T82.818A	Embolism due to vascular prosthetic devices, implants and grafts, initial encounter
I26.01	Septic pulmonary embolism with acute cor pulmonale
I26.90	Septic pulmonary embolism without acute cor pulmonale
I26.02	Saddle embolus of pulmonary artery with acute cor pulmonale
I26.92	Saddle embolus of pulmonary artery without acute cor pulmonale
I2693	Single subsegmental pulmonary embolism without acute cor pulmonale
I2694	Multiple subsegmental pulmonary emboli without acute cor pulmonale
I26.09	Other pulmonary embolism and infarction with acute cor pulmonale
I26.99	Other pulmonary embolism and infarction without acute cor pulmonale
I80.10	Phlebitis and thrombophlebitis of unspecified femoral vein

I80.11	Phlebitis and thrombophlebitis of right femoral vein
I80.12	Phlebitis and thrombophlebitis of left femoral vein
I80.13	Phlebitis and thrombophlebitis of bilateral femoral vein
I80.209	Phlebitis and thrombophlebitis of deep veins of lower extremities of unspecified lower extremity
I80.201	Phlebitis and thrombophlebitis of deep veins of lower extremities of right lower extremity
I80.202	Phlebitis and thrombophlebitis of deep veins of lower extremities of left lower extremity
I80.203	Phlebitis and thrombophlebitis of deep veins of lower extremities of bilateral lower extremity
I80.229	Phlebitis and thrombophlebitis of unspecified popliteal vein
I80.221	Phlebitis and thrombophlebitis of right popliteal vein
I80.222	Phlebitis and thrombophlebitis of left popliteal vein
I80.223	Phlebitis and thrombophlebitis of bilateral popliteal vein
I80.239	Phlebitis and thrombophlebitis of unspecified tibial vein
I80.231	Phlebitis and thrombophlebitis of unspecified right vein
I80.232	Phlebitis and thrombophlebitis of unspecified left vein
I80.233	Phlebitis and thrombophlebitis of bilateral tibial vein
I80.299	Phlebitis and thrombophlebitis of other deep vessels of unspecified lower extremity
I80.291	Phlebitis and thrombophlebitis of other deep vessels of right lower extremity
I80.292	Phlebitis and thrombophlebitis of other deep vessels of left lower extremity
I80.293	Phlebitis and thrombophlebitis of other deep vessels of bilateral lower extremity
I80.3	Phlebitis and thrombophlebitis of lower extremities, unspecified
I80.211	Phlebitis and thrombophlebitis of right iliac vein
I80.212	Phlebitis and thrombophlebitis of left iliac vein
I80.213	Phlebitis and thrombophlebitis of iliac vein, bilateral
I80.219	Phlebitis and thrombophlebitis of unspecified iliac vein
I82.611	Acute embolism and thrombosis of superficial veins of right upper extremity
I82.612	Acute embolism and thrombosis of superficial veins of left upper extremity
I82.613	Acute embolism and thrombosis of superficial veins of upper extremity, bilateral
I82.619	Acute embolism and thrombosis of superficial veins of unspecified upper extremity
I82.621	Acute embolism and thrombosis of deep veins of right upper extremity
I82.622	Acute embolism and thrombosis of deep veins of left upper extremity
I82.623	Acute embolism and thrombosis of deep veins of upper extremity, bilateral
I82.629	Acute embolism and thrombosis of deep veins of unspecified upper extremity
I82.601	Acute embolism and thrombosis of unspecified veins of right upper extremity
I82.602	Acute embolism and thrombosis of unspecified veins of left upper extremity
I82.603	Acute embolism and thrombosis of unspecified veins of upper extremity, bilateral
I82.609	Acute embolism and thrombosis of unspecified veins of unspecified upper extremity
I82.A11	Acute embolism and thrombosis of right axillary vein
I82.A12	Acute embolism and thrombosis of left axillary vein
I82.A13	Acute embolism and thrombosis of axillary vein, bilateral
I82.A19	Acute embolism and thrombosis of unspecified axillary vein
I82.B11	Acute embolism and thrombosis of right subclavian vein
I82.B12	Acute embolism and thrombosis of left subclavian vein

I82.B13	Acute embolism and thrombosis of subclavian vein, bilateral
I82.B19	Acute embolism and thrombosis of unspecified subclavian vein
I82.C11	Acute embolism and thrombosis of right internal jugular vein
I82.C12	Acute embolism and thrombosis of left internal jugular vein
I82.C13	Acute embolism and thrombosis of internal jugular vein, bilateral
I82.C19	Acute embolism and thrombosis of unspecified internal jugular vein
I82.210	Acute embolism and thrombosis of superior vena cava
I82.290	Acute embolism and thrombosis of other thoracic veins
I82.890	Acute embolism and thrombosis of other specified veins
I82.90	Acute embolism and thrombosis of unspecified vein
J13.	Pneumonia due to Streptococcus pneumoniae
J15.0	Pneumonia due to Klebsiella pneumoniae
J15.1	Pneumonia due to Pseudomonas
J15.4	Pneumonia due to other streptococci
J15.4	Pneumonia due to other streptococci
J15.3	Pneumonia due to streptococcus, group B
J15.4	Pneumonia due to other streptococci
J15.20	Pneumonia due to staphylococcus, unspecified
J15.29	Pneumonia due to other staphylococcus
J15.8	Pneumonia due to other specified bacteria
A48.1	Legionnaires' disease
J15.8	Pneumonia due to other specified bacteria
J15.9	Unspecified bacterial pneumonia
J15.7	Pneumonia due to Mycoplasma pneumoniae
B25.0	Cytomegaloviral pneumonitis
A37.01	Whooping cough due to Bordetella pertussis with pneumonia
A37.11	Whooping cough due to Bordetella parapertussis with pneumonia
A37.91	Whooping cough, unspecified species with pneumonia
A22.1	Pulmonary anthrax
B44.0	Invasive pulmonary aspergillosis
J17.	Pneumonia in diseases classified elsewhere
B77.81	Ascariasis pneumonia
J17.	Pneumonia in diseases classified elsewhere
J18.0	Bronchopneumonia, unspecified organism
J69.0	Pneumonitis due to inhalation of food and vomit
J81.0	Acute pulmonary edema
J95811	Postprocedural pneumothorax
J95812	Postprocedural air leak
J95.821	Acute postprocedural respiratory failure
J96.00	Acute respiratory failure, unspecified whether with hypoxia or hypercapnia
J96.01	Acute respiratory failure with hypoxia

J96.02	Acute respiratory failure with hypercapnia
J95.1	Acute pulmonary insufficiency following thoracic surgery
J95.2	Acute pulmonary insufficiency following nonthoracic surgery
J95.3	Chronic pulmonary insufficiency following surgery
J95.822	Acute and chronic postprocedural respiratory failure
J96.20	Acute and chronic respiratory failure, unspecified whether with hypoxia or hypercapnia
J96.00	Acute respiratory failure, unspecified whether with hypoxia or hypercapnia
J96.01	Acute respiratory failure with hypoxia
J96.02	Acute respiratory failure with hypercapnia
J96.90	Respiratory failure, unspecified, unspecified whether with hypoxia or hypercapnia
J96.91	Respiratory failure, unspecified with hypoxia
J96.92	Respiratory failure, unspecified with hypercapnia
J80.	Acute respiratory distress syndrome
R06.03	Acute respiratory distress
J96.10	Chronic respiratory failure, unspecified whether with hypoxia or hypercapnia
J96.11	Chronic respiratory failure with hypoxia
J96.12	Chronic respiratory failure with hypercapnia
J96.20	Acute and chronic respiratory failure, unspecified whether with hypoxia or hypercapnia
J96.21	Acute and chronic respiratory failure with hypoxia
J96.22	Acute and chronic respiratory failure with hypercapnia
J98.4	Other disorders of lung
K25.0	Acute gastric ulcer with hemorrhage
K56.699	Other intestinal obstruction unspecified as to partial versus complete obstruction
K25.1	Acute gastric ulcer with perforation
K25.2	Acute gastric ulcer with both hemorrhage and perforation
K56.699	Other intestinal obstruction unspecified as to partial versus complete obstruction
K25.4	Chronic or unspecified gastric ulcer with hemorrhage
K25.6	Chronic or unspecified gastric ulcer with both hemorrhage and perforation
K26.0	Acute duodenal ulcer with hemorrhage
K26.1	Acute duodenal ulcer with perforation
K26.2	Acute duodenal ulcer with both hemorrhage and perforation
K26.4	Chronic or unspecified duodenal ulcer with hemorrhage
K26.6	Chronic or unspecified duodenal ulcer with both hemorrhage and perforation
K27.0	Acute peptic ulcer, site unspecified, with hemorrhage
K27.1	Acute peptic ulcer, site unspecified, with perforation
K27.2	Acute peptic ulcer, site unspecified, with both hemorrhage and perforation
K27.4	Chronic or unspecified peptic ulcer, site unspecified, with hemorrhage
K27.6	Chronic or unspecified peptic ulcer, site unspecified, with both hemorrhage and perforation
K28.0	Acute gastrojejunal ulcer with hemorrhage
K28.1	Acute gastrojejunal ulcer with perforation
K28.2	Acute gastrojejunal ulcer with both hemorrhage and perforation

K28.4	Chronic or unspecified gastrojejunal ulcer with hemorrhage
K28.6	Chronic or unspecified gastrojejunal ulcer with both hemorrhage and perforation
K29.01	Acute gastritis with bleeding
K29.31	Chronic superficial gastritis with bleeding
K29.41	Chronic atrophic gastritis with bleeding
K29.51	Unspecified chronic gastritis with bleeding
K29.61	Other gastritis with bleeding
K29.21	Alcoholic gastritis with bleeding
K29.31	Chronic superficial gastritis with bleeding
K29.71	Gastritis, unspecified, with bleeding
K29.91	Gastroduodenitis, unspecified, with bleeding
K29.81	Duodenitis with bleeding
K92.2	Gastrointestinal hemorrhage, unspecified
N170	Acute kidney failure with tubular necrosis
N171	Acute kidney failure with acute cortical necrosis
N172	Acute kidney failure with medullary necrosis
N178	Other acute kidney failure
N17.9	Acute kidney failure, unspecified
N990	Postprocedural (acute) (chronic) kidney failure
D78.01	Intraoperative hemorrhage and hematoma of the spleen complicating a procedure on the spleen
D78.02	Intraoperative hemorrhage and hematoma of the spleen complicating other procedure
D78.21	Postprocedural hemorrhage of the spleen following a procedure on the spleen
D78.22	Postprocedural hemorrhage of the spleen following other procedure
E36.01	Intraoperative hemorrhage and hematoma of an endocrine system organ or structure complicating an endocrine system procedure
E36.02	Intraoperative hemorrhage and hematoma of an endocrine system organ or structure complicating other procedure
E89.810	Postprocedural hemorrhage of an endocrine system organ or structure following an endocrine system procedure
E89.811	Postprocedural hemorrhage of an endocrine system organ or structure following other procedure
G97.31	Intraoperative hemorrhage and hematoma of a nervous system organ or structure complicating a nervous system procedure
G97.32	Intraoperative hemorrhage and hematoma of a nervous system organ or structure complicating other procedure
G97.51	Postprocedural hemorrhage of a nervous system organ or structure following a nervous system procedure
G97.52	Postprocedural hemorrhage of a nervous system organ or structure following other procedure
H59.111	Intraoperative hemorrhage and hematoma of right eye and adnexa complicating an ophthalmic procedure
H59.112	Intraoperative hemorrhage and hematoma of left eye and adnexa complicating an ophthalmic procedure
H59.113	Intraoperative hemorrhage and hematoma of eye and adnexa complicating an ophthalmic procedure, bilateral
H59.119	Intraoperative hemorrhage and hematoma of unspecified eye and adnexa complicating an ophthalmic procedure
H59.121	Intraoperative hemorrhage and hematoma of right eye and adnexa complicating other procedure
H59.122	Intraoperative hemorrhage and hematoma of left eye and adnexa complicating other procedure

H59.123	Intraoperative hemorrhage and hematoma of eye and adnexa complicating other procedure, bilateral
H59.129	Intraoperative hemorrhage and hematoma of unspecified eye and adnexa complicating other procedure
H59.311	Postprocedural hemorrhage of right eye and adnexa following an ophthalmic procedure
H59.312	Postprocedural hemorrhage of left eye and adnexa following an ophthalmic procedure
H59.313	Postprocedural hemorrhage of eye and adnexa following an ophthalmic procedure, bilateral
H59.319	Postprocedural hemorrhage of unspecified eye and adnexa following an ophthalmic procedure
H59.321	Postprocedural hemorrhage of right eye and adnexa following other procedure
H59.322	Postprocedural hemorrhage of left eye and adnexa following other procedure
H59.323	Postprocedural hemorrhage of eye and adnexa following other procedure, bilateral
H59.329	Postprocedural hemorrhage of unspecified eye and adnexa following other procedure
H95.21	Intraoperative hemorrhage and hematoma of ear and mastoid process complicating a procedure on the ear and mastoid process
H95.22	Intraoperative hemorrhage and hematoma of ear and mastoid process complicating other procedure
H95.41	Postprocedural hemorrhage of ear and mastoid process following a procedure on the ear and mastoid process
H95.42	Postprocedural hemorrhage of ear and mastoid process following other procedure
I97.410	Intraoperative hemorrhage and hematoma of a circulatory system organ or structure complicating a cardiac catheterization
I97.411	Intraoperative hemorrhage and hematoma of a circulatory system organ or structure complicating a cardiac bypass
I97.418	Intraoperative hemorrhage and hematoma of a circulatory system organ or structure complicating other circulatory system procedure
I97.42	Intraoperative hemorrhage and hematoma of a circulatory system organ or structure complicating other procedure
I97.610	Postprocedural hemorrhage of a circulatory system organ or structure following a cardiac catheterization
I97.611	Postprocedural hemorrhage of a circulatory system organ or structure following cardiac bypass
I97.618	Postprocedural hemorrhage of a circulatory system organ or structure following other circulatory system procedure
I97.620	Postprocedural hemorrhage of a circulatory system organ or structure following other procedure
J95.61	Intraoperative hemorrhage and hematoma of a respiratory system organ or structure complicating a respiratory system procedure
J95.62	Intraoperative hemorrhage and hematoma of a respiratory system organ or structure complicating other procedure
J95.830	Postprocedural hemorrhage of a respiratory system organ or structure following a respiratory system procedure
J95.831	Postprocedural hemorrhage of a respiratory system organ or structure following other procedure
J95861	Postprocedural hematoma of a respiratory system organ or structure following other procedure
J95862	Postprocedural seroma of a respiratory system organ or structure following a respiratory system procedure
J95863	Postprocedural seroma of a respiratory system organ or structure following other procedure
K91.61	Intraoperative hemorrhage and hematoma of a digestive system organ or structure complicating a digestive system procedure
K91.62	Intraoperative hemorrhage and hematoma of a digestive system organ or structure complicating other procedure
K91.840	Postprocedural hemorrhage of a digestive system organ or structure following a digestive system procedure
K91.841	Postprocedural hemorrhage of a digestive system organ or structure following other procedure

L76.01	Intraoperative hemorrhage and hematoma of skin and subcutaneous tissue complicating a dermatologic procedure
L76.02	Intraoperative hemorrhage and hematoma of skin and subcutaneous tissue complicating other procedure
L76.21	Postprocedural hemorrhage of skin and subcutaneous tissue following a dermatologic procedure
L76.22	Postprocedural hemorrhage of skin and subcutaneous tissue following other procedure
M96.810	Intraoperative hemorrhage and hematoma of a musculoskeletal structure complicating a musculoskeletal system procedure
M96.811	Intraoperative hemorrhage and hematoma of a musculoskeletal structure complicating other procedure
M96.830	Postprocedural hemorrhage of a musculoskeletal structure following a musculoskeletal system procedure
M96.831	Postprocedural hemorrhage of a musculoskeletal structure following other procedure
N99.61	Intraoperative hemorrhage and hematoma of a genitourinary system organ or structure complicating a genitourinary system procedure
N99.62	Intraoperative hemorrhage and hematoma of a genitourinary system organ or structure complicating other procedure
N99.820	Postprocedural hemorrhage of a genitourinary system organ or structure following a genitourinary system procedure
N99.821	Postprocedural hemorrhage of a genitourinary system organ or structure following other procedure
D78.01	Intraoperative hemorrhage and hematoma of the spleen complicating a procedure on the spleen
D78.02	Intraoperative hemorrhage and hematoma of the spleen complicating other procedure
D78.31	Postprocedural hematoma of the spleen following a procedure on the spleen
D78.32	Postprocedural hematoma of the spleen following other procedure
E36.01	Intraoperative hemorrhage and hematoma of an endocrine system organ or structure complicating an endocrine system procedure
E36.02	Intraoperative hemorrhage and hematoma of an endocrine system organ or structure complicating other procedure
E89.820	Postprocedural hematoma of an endocrine system organ or structure following an endocrine system procedure
E89.821	Postprocedural hematoma of an endocrine system organ or structure following other procedure
G97.31	Intraoperative hemorrhage and hematoma of a nervous system organ or structure complicating a nervous system procedure
G97.32	Intraoperative hemorrhage and hematoma of a nervous system organ or structure complicating other procedure
G97.61	Postprocedural hematoma of a nervous system organ or structure following a nervous system procedure
G97.62	Postprocedural hematoma of a nervous system organ or structure following other procedure
H59.111	Intraoperative hemorrhage and hematoma of right eye and adnexa complicating an ophthalmic procedure
H59.112	Intraoperative hemorrhage and hematoma of left eye and adnexa complicating an ophthalmic procedure
H59.113	Intraoperative hemorrhage and hematoma of eye and adnexa complicating an ophthalmic procedure, bilateral
H59.119	Intraoperative hemorrhage and hematoma of unspecified eye and adnexa complicating an ophthalmic procedure
H59.121	Intraoperative hemorrhage and hematoma of right eye and adnexa complicating other procedure
H59.122	Intraoperative hemorrhage and hematoma of left eye and adnexa complicating other procedure
H59.123	Intraoperative hemorrhage and hematoma of eye and adnexa complicating other procedure, bilateral
H59.129	Intraoperative hemorrhage and hematoma of unspecified eye and adnexa complicating other procedure
H59.331	Postprocedural hematoma of right eye and adnexa following an ophthalmic procedure
H59.332	Postprocedural hematoma of left eye and adnexa following an ophthalmic procedure

H59.333	Postprocedural hematoma of eye and adnexa following an ophthalmic procedure, bilateral
H59.339	Postprocedural hematoma of unspecified eye and adnexa following an ophthalmic procedure
H59.341	Postprocedural hematoma of right eye and adnexa following other procedure
H59.342	Postprocedural hematoma of left eye and adnexa following other procedure
H59.343	Postprocedural hematoma of eye and adnexa following other procedure, bilateral
H59.349	Postprocedural hematoma of unspecified eye and adnexa following other procedure
H95.21	Intraoperative hemorrhage and hematoma of ear and mastoid process complicating a procedure on the ear and mastoid process
H95.22	Intraoperative hemorrhage and hematoma of ear and mastoid process complicating other procedure
H95.51	Postprocedural hematoma of ear and mastoid process following a procedure on the ear and mastoid process
H95.52	Postprocedural hematoma of ear and mastoid process following other procedure
I97.410	Intraoperative hemorrhage and hematoma of a circulatory system organ or structure complicating a cardiac catheterization
I97.411	Intraoperative hemorrhage and hematoma of a circulatory system organ or structure complicating a cardiac bypass
I97.418	Intraoperative hemorrhage and hematoma of a circulatory system organ or structure complicating other circulatory system procedure
I97.42	Intraoperative hemorrhage and hematoma of a circulatory system organ or structure complicating other procedure
I97.621	Postprocedural hematoma of a circulatory system organ or structure following other procedure
I97.630	Postprocedural hematoma of a circulatory system organ or structure following a cardiac catheterization
I97.631	Postprocedural hematoma of a circulatory system organ or structure following cardiac bypass
I97.638	Postprocedural hematoma of a circulatory system organ or structure following other circulatory system procedure
J95.61	Intraoperative hemorrhage and hematoma of a respiratory system organ or structure complicating a respiratory system procedure
J95.62	Intraoperative hemorrhage and hematoma of a respiratory system organ or structure complicating other procedure
J95.860	Postprocedural hematoma of a respiratory system organ or structure following a respiratory system procedure
J95.861	Postprocedural hematoma of a respiratory system organ or structure following other procedure
K91.61	Intraoperative hemorrhage and hematoma of a digestive system organ or structure complicating a digestive system procedure
K91.62	Intraoperative hemorrhage and hematoma of a digestive system organ or structure complicating other procedure
K91.870	Postprocedural hematoma of a digestive system organ or structure following a digestive system procedure
K91.871	Postprocedural hematoma of a digestive system organ or structure following other procedure
L76.01	Intraoperative hemorrhage and hematoma of skin and subcutaneous tissue complicating a dermatologic procedure
L76.02	Intraoperative hemorrhage and hematoma of skin and subcutaneous tissue complicating other procedure
L76.31	Postprocedural hematoma of skin and subcutaneous tissue following a dermatologic procedure
L76.32	Postprocedural hematoma of skin and subcutaneous tissue following other procedure
M96.810	Intraoperative hemorrhage and hematoma of a musculoskeletal structure complicating a musculoskeletal system procedure
M96.811	Intraoperative hemorrhage and hematoma of a musculoskeletal structure complicating other procedure
M96.840	Postprocedural hematoma of a musculoskeletal structure following a musculoskeletal system procedure

M96.841	Postprocedural hematoma of a musculoskeletal structure following other procedure
N99.61	Intraoperative hemorrhage and hematoma of a genitourinary system organ or structure complicating a genitourinary system procedure
N99.62	Intraoperative hemorrhage and hematoma of a genitourinary system organ or structure complicating other procedure
N99.840	Postprocedural hematoma of a genitourinary system organ or structure following a genitourinary system procedure
N99.841	Postprocedural hematoma of a genitourinary system organ or structure following other procedure
D78.33	Postprocedural seroma of the spleen following a procedure on the spleen
D78.34	Postprocedural seroma of the spleen following other procedure
E89.822	Postprocedural seroma of an endocrine system organ or structure following an endocrine system procedure
E89.823	Postprocedural seroma of an endocrine system organ or structure following other procedure
G97.63	Postprocedural seroma of a nervous system organ or structure following a nervous system procedure
G97.64	Postprocedural seroma of a nervous system organ or structure following other procedure
H59.351	Postprocedural seroma of right eye and adnexa following an ophthalmic procedure
H59.352	Postprocedural seroma of left eye and adnexa following an ophthalmic procedure
H59.353	Postprocedural seroma of eye and adnexa following an ophthalmic procedure, bilateral
H59.359	Postprocedural seroma of unspecified eye and adnexa following an ophthalmic procedure
H59.361	Postprocedural seroma of right eye and adnexa following other procedure
H59.362	Postprocedural seroma of left eye and adnexa following other procedure
H59.363	Postprocedural seroma of eye and adnexa following other procedure, bilateral
H59.369	Postprocedural seroma of unspecified eye and adnexa following other procedure
H95.53	Postprocedural seroma of ear and mastoid process following a procedure on the ear and mastoid process
H95.54	Postprocedural seroma of ear and mastoid process following other procedure
I9788	Other intraoperative complications of the circulatory system, not elsewhere classified
I9789	Other postprocedural complications and disorders of the circulatory system, not elsewhere classified
I97.622	Postprocedural seroma of a circulatory system organ or structure following other procedure
I97.640	Postprocedural seroma of a circulatory system organ or structure following a cardiac catheterization
I97.641	Postprocedural seroma of a circulatory system organ or structure following cardiac bypass
I97.648	Postprocedural seroma of a circulatory system organ or structure following other circulatory system procedure
J95.862	Postprocedural seroma of a respiratory system organ or structure following a respiratory system procedure
J95.863	Postprocedural seroma of a respiratory system organ or structure following other procedure
K91.872	Postprocedural seroma of a digestive system organ or structure following a digestive system procedure
K91.873	Postprocedural seroma of a digestive system organ or structure following other procedure
L76.33	Postprocedural seroma of skin and subcutaneous tissue following a dermatologic procedure
L76.34	Postprocedural seroma of skin and subcutaneous tissue following other procedure
M96.842	Postprocedural seroma of a musculoskeletal structure following a musculoskeletal system procedure
M96.843	Postprocedural seroma of a musculoskeletal structure following other procedure
N99.842	Postprocedural seroma of a genitourinary system organ or structure following a genitourinary system procedure
N99.843	Postprocedural seroma of a genitourinary system organ or structure following other procedure
T88.8XXA	Other specified complications of surgical and medical care, not elsewhere classified, initial encounter

T81.30XA	Disruption of wound, unspecified, initial encounter
T81.32XA	Disruption of internal operation (surgical) wound, not elsewhere classified, initial encounter
T81.31XA	Disruption of external operation (surgical) wound, not elsewhere classified, initial encounter
T81.33XA	Disruption of traumatic injury wound repair, initial encounter
T8140XA	Infection following a procedure, unspecified, initial encounter
T8140XD	Infection following a procedure, unspecified, subsequent encounter
T8140XS	Infection following a procedure, unspecified, sequela
T8141XA	Infection following a procedure, superficial incisional surgical site, initial encounter
T8141XD	Infection following a procedure, superficial incisional surgical site, subsequent encounter
T8141XS	Infection following a procedure, superficial incisional surgical site, sequela
T8142XA	Infection following a procedure, deep incisional surgical site, initial encounter
T8142XD	Infection following a procedure, deep incisional surgical site, subsequent encounter
T8142XS	Infection following a procedure, deep incisional surgical site, sequela
T8143XA	Infection following a procedure, organ and space surgical site, initial encounter
T8143XD	Infection following a procedure, organ and space surgical site, subsequent encounter
T8143XS	Infection following a procedure, organ and space surgical site, sequela
T8144XA	Sepsis following a procedure, initial encounter
T8144XD	Sepsis following a procedure, subsequent encounter
T8144XS	Sepsis following a procedure, sequela
T8149XA	Infection following a procedure, other surgical site, initial encounter
T8149XD	Infection following a procedure, other surgical site, subsequent encounter
T8149XS	Infection following a procedure, other surgical site, sequela
T8110XA	Postprocedural shock unspecified, initial encounter
T8110XD	Postprocedural shock unspecified, subsequent encounter
T8110XS	Postprocedural shock unspecified, sequela
T8111XA	Postprocedural cardiogenic shock, initial encounter
T8111XD	Postprocedural cardiogenic shock, subsequent encounter
T8111XS	Postprocedural cardiogenic shock, sequela
T8112XA	Postprocedural septic shock, initial encounter
T8112XD	Postprocedural septic shock, subsequent encounter
T8112XS	Postprocedural septic shock, sequela
T8119XA	Other postprocedural shock, initial encounter
T8119XD	Other postprocedural shock, subsequent encounter
T8119XS	Other postprocedural shock, sequela
A400	Sepsis due to streptococcus, group A
A401	Sepsis due to streptococcus, group B
A403	Sepsis due to Streptococcus pneumoniae
A408	Other streptococcal sepsis
A409	Streptococcal sepsis, unspecified
A4101	Sepsis due to Methicillin susceptible Staphylococcus aureus
A4102	Sepsis due to Methicillin resistant Staphylococcus aureus
A411	Sepsis due to other specified staphylococcus
A412	Sepsis due to unspecified staphylococcus

A413	Sepsis due to Hemophilus influenzae
A414	Sepsis due to anaerobes
A4150	Gram-negative sepsis, unspecified
A4151	Sepsis due to Escherichia coli [E. coli]
A4152	Sepsis due to Pseudomonas
A4153	Sepsis due to Serratia
A4159	Other Gram-negative sepsis
A4181	Sepsis due to Enterococcus
A4189	Other specified sepsis
A419	Sepsis, unspecified organism
K68.11	Postprocedural retroperitoneal abscess
K68.19	Other retroperitoneal abscess
T81.4XXA	Infection following a procedure, initial encounter
G97.2	Intracranial hypotension following ventricular shunting
G97.81	Other intraoperative complications of nervous system
G97.82	Other postprocedural complications and disorders of nervous system
G97.31	Intraoperative hemorrhage and hematoma of a nervous system organ or structure complicating a nervous system procedure
G97.32	Intraoperative hemorrhage and hematoma of a nervous system organ or structure complicating other procedure
I97.810	Intraoperative cerebrovascular infarction during cardiac surgery
I97.811	Intraoperative cerebrovascular infarction during other surgery
I97.820	Postprocedural cerebrovascular infarction following cardiac surgery
I97.821	Postprocedural cerebrovascular infarction following other surgery
J95.851	Ventilator associated pneumonia
J95.89	Other postprocedural complications and disorders of respiratory system, not elsewhere classified
J95.4	Chemical pneumonitis due to anesthesia
J95.5	Postprocedural subglottic stenosis
J95.859	Other complication of respirator [ventilator]
J95.88	Other intraoperative complications of respiratory system, not elsewhere classified
J95.89	Other postprocedural complications and disorders of respiratory system, not elsewhere classified
N39.0	Urinary tract infection, site not specified
N30.00	Acute cystitis without hematuria
N30.01	Acute cystitis with hematuria
N41.3	Prostatocystitis
N10	Acute pyelonephritis
N15.1	Renal and perinephric abscess
N34.0	Uretheral abscess
N34.2	Other urethritis
I46.2	Cardiac arrest due to underlying cardiac condition
I46.8	Cardiac arrest due to other underlying condition
I46.9	Cardiac arrest, cause unspecified
I97.710	Intraoperative cardiac arrest during cardiac surgery

I97.711	Intraoperative cardiac arrest during other surgery
R09.2	Cardiorespiratory arrest
I97.810	Intraoperative cerebrovascular infarction during cardiac surgery
I97.811	Intraoperative cerebrovascular infarction during other surgery
I97.820	Postprocedural cerebrovascular infarction following cardiac surgery
I97.821	Postprocedural cerebrovascular infarction following other surgery
I97111	Postprocedural cardiac insufficiency following other surgery
F05	Delirium due to known physiological condition
R41.0	Disorientation, unspecified

Supplementary Table 4. Adjusted outcomes after open and endovascular abdominal aortic aneurysm, stratified by admission status

	Elective (N=37,019)		Urgent/Emergent (N=5,714)	
	Adjusted ORs	p-value	Adjusted ORs	p-value
Inpatient Death	NA		0.98 (0.61 – 1.56)	0.93
30-Day Death	1.35 (1.01 – 1.79)	0.04	1.35 (0.95 – 1.90)	0.09
90-Day Death	1.52 (1.23 – 1.88)	<.001	1.20 (0.90 – 1.60)	0.21
1-Year Death[1]	1.86 (1.61 – 2.14)	<.001	1.36 (1.06 – 1.74)	0.02
Inpatient Complication	1.12 (0.96 – 1.32)	0.15	1.03 (0.80 – 1.32)	0.82
30-Day Complication[2]	1.24 (1.08 – 1.43)	0.003	1.00 (0.77 – 1.28)	0.97
Discharge to Higher Level of Care[3]	2.82 (2.42 – 3.30)	<.001	1.98 (1.53 – 2.56)	<.001
Discharge to Home[4]	0.48 (0.40 – 0.58)	<.001	0.45 (0.33 – 0.60)	<.001
30-Day Readmission[2]	1.24 (1.06 – 1.46)	0.008	0.96 (0.74 – 1.26)	0.77
90-Day Readmission[2]	1.21 (1.06 – 1.39)	0.006	0.91 (0.72 – 1.15)	0.42
90-Day Any Intensive Intervention[2]	1.11 (0.80 – 1.56)	0.53	0.97 (0.63 – 1.51)	0.42
	Adjusted Mean Difference		Adjusted Mean Difference	
Hospital Length of Stay	0.18 (-0.14 – 0.51)	0.26	0.08 (-0.58 – 0.75)	0.80
Home-time Ratio[3]	-0.06 (-0.08 – -0.05)	<.001	-0.08 (-0.13 – -0.04)	<.001

[1] Patients who underwent index surgery before 1/1/2020 and have continuous Medicare FFS enrollment for 12 months after discharge.

[2] Patients who died within the follow-up time frame and without event of interest were removed from analysis.

[3] Patients admitted from the highest level of care (LTAC) were removed from analysis.

[4] Analysis restricted to community-dwelling patients with index admission date between 1/1/2017 to 12/31/2018.

[5] Analysis restricted to community-dwelling patients and discharged before 10/1/2018. And patients who died within the follow-up time frame and without event of interest were removed from analysis.
